# A benzamide‐dependent *fts* *Z* mutant reveals residues crucial for Z‐ring assembly

**DOI:** 10.1111/mmi.13286

**Published:** 2015-12-22

**Authors:** David William Adams, Ling Juan Wu, Jeff Errington

**Affiliations:** ^1^ Centre for Bacterial Cell BiologyBaddiley‐Clark BuildingMedical SchoolNewcastle UniversityRichardson RoadNewcastle upon TyneNE2 4AXUK; ^2^ Present address: Laboratory of Molecular MicrobiologyGlobal Health InstituteSchool of Life SciencesEcole Polytechnique Fédérale de LausanneLausanneCH‐1015Switzerland

## Abstract

In almost all bacteria, cell division is co‐ordinated by the essential tubulin homologue FtsZ and represents an attractive but as yet unexploited target for new antibiotics. The benzamides, e.g. PC190723, are potent FtsZ inhibitors that have the potential to yield an important new class of antibiotic. However, the evolution of resistance poses a challenge to their development. Here we show that a collection of PC190723‐resistant and ‐dependent strains of *S* *taphylococcus aureus* exhibit severe growth and morphological defects, questioning whether these *fts* *Z* mutations would be clinically relevant. Importantly, we show that the most commonly isolated substitution remains sensitive to the simplest benzamide 3‐MBA and likely works by occluding compound binding. Extending this analysis to *B* *acillus subtilis*, we isolated a novel benzamide‐dependent strain that divides using unusual helical division events. The *fts* *Z* mutation responsible encodes the substitution of a highly conserved residue, which lies outside the benzamide‐binding site and forms part of an interface between the N‐ and C‐terminal domains that we show is necessary for normal FtsZ function. Together with an intragenic suppressor mutation that mimics benzamide binding, the results provide genetic evidence that benzamides restrict conformational changes in FtsZ and also highlights their utility as tools to probe bacterial division.

## Introduction

The ongoing proliferation of antibiotic resistance among important bacterial pathogens presents a global threat to human health and necessitates the development of new antibiotics with novel modes of action. One attractive, but as yet clinically unexploited target, is bacterial cell division, which in most bacteria, is orchestrated by the tubulin ancestor FtsZ. FtsZ is a self‐assembling GTPase that upon GTP‐binding assembles cooperatively into single‐stranded proto‐filaments via the head to tail association of individual subunits, and at the onset of division forms a ring‐like structure, known as the Z‐ring (Bi and Lutkenhaus, 1991; Erickson *et al*., 2010). Although the precise architecture of the Z‐ring remains unclear (Holden *et al*., 2014; Meier and Goley, 2014; Szwedziak *et al*., 2014), it is generally accepted that it co‐ordinates division by acting as a dynamic framework for assembly of the divisome; a large multi‐protein complex that, in addition to various structural and regulatory components, contains the cell wall synthetic machinery that are necessary for septum synthesis (Adams and Errington, 2009; Egan and Vollmer, 2013). Notably, the demonstration that inhibiting cell division in the important human pathogen *Staphylococcus aureus* leads to a rapid loss in viability highlighted the potential of cell division inhibitors to act as bactericidal antibiotics (Pinho and Errington, 2003; Stokes *et al*., 2005). Indeed, much effort has been dedicated to identifying inhibitors of FtsZ, and a diverse range of compounds have been reported to inhibit its assembly *in vitro* as well as some that inhibit cell division *in vivo* (Schaffner‐Barbero *et al*., 2012). However, for most of these compounds, little is known about the mode of action or the binding site and indeed some have even turned out to act indirectly (Foss *et al*., 2013).

**Figure 1:**
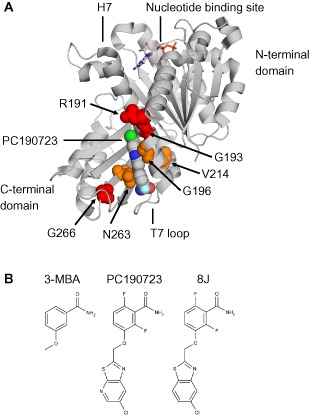
The benzamide binding site. A. Cartoon representation of *S*. *aureus* FtsZ‐PC190723‐GDP complex (PDB 3VOB). GDP and PC190723 (PC) are coloured according to chemical element. PC and residues altered by *fts* *Z* mutations are shown as spheres. PC‐resistant substitutions are shown in orange; PC‐dependent substitutions are shown in red. B. Chemical structures of 3‐methoxybenzamide (3‐MBA), PC190723 and 8J.

The best‐studied FtsZ inhibitors are the benzamides, a family of synthetic 3‐methoxybenzamide (3‐MBA) derivatives, exemplified by compounds PC190723 (PC) and 8J (Fig. 1B). The benzamides exhibit potent, on‐target antibacterial activity against a subset of Gram‐positive bacteria and are the only class of FtsZ inhibitor shown to be efficacious in murine models of infection (Ohashi *et al*., 1999; Haydon *et al*., 2008; 2010; Czaplewski *et al*., 2009). *In vivo*, the benzamides block Z‐ring assembly, and thus division, by promoting the mislocalisation of FtsZ into multiple dynamic but non‐productive foci (Haydon *et al*., 2008; Adams *et al*., 2011). They appear to act by blocking one or more steps of the normal FtsZ assembly cycle and *in vitro* enhance FtsZ assembly in a GTP‐dependent manner, resulting in the formation of unusually stable polymers that are resistant to GDP‐induced disassembly (Andreu *et al*., 2010; Adams *et al*., 2011). Under conditions that facilitate FtsZ assembly, these polymers often consist of large multi‐stranded structures, with low GTPase activity, whereas under more physiologically relevant conditions, GTPase activity is unaffected and the polymers remain single‐stranded but are highly curved (Andreu *et al*., 2010; Adams *et al*., 2011).

FtsZ consists of two major globular domains separated by a central core helix (H7) (Fig. 1A) (Löwe and Amos, 1998). The polymerisation interface is formed by the insertion of the T7 loop, which contains catalytic residues required for GTP hydrolysis, from the base of H7 of one subunit into the nucleotide binding site of the subunit below (Löwe and Amos, 1999; Scheffers *et al*., 2002; Oliva *et al*., 2004). Unlike most other FtsZ inhibitors, the benzamide binding site is distinct from that of the nucleotide and away from the polymerisation interface (Haydon *et al*., 2008). Indeed, recent co‐crystal structures of PC190723 and *S. aureus* FtsZ have confirmed that the benzamides bind within a deep hydrophobic cleft formed between the C‐terminal domain, H7 and the T7 loop (Fig. 1A) (Matsui *et al*., 2012; Tan *et al*., 2012). Interestingly, this cleft likely transitions between the open and closed states throughout the FtsZ assembly cycle and may reflect assembly competent and incompetent conformations respectively (Elsen *et al*., 2012; Matsui *et al*., 2012), in line with previous predictions that a conformational change upon polymerisation drives cooperative assembly (Dajkovic *et al*., 2008; Huecas *et al*., 2008; Lan *et al*., 2008; Miraldi *et al*., 2008). Benzamide binding would therefore maintain the cleft in the open conformation and so drive the system towards assembly (Elsen *et al*., 2012; Matsui *et al*., 2012; Ramírez‐Aportela *et al*., 2014).

Haydon *et al*. have previously isolated spontaneously arising *ftsZ* mutants that rendered *S. aureus* non‐susceptible to PC190723. Each mutation encodes an amino acid substitution at one of six different residues that sit in and around the benzamide binding site (Fig. 1A). Intriguingly, the phenotypes of these mutants segregated into two distinct classes, drug‐resistant (G196A, V214F and N263K) and drug‐dependent (R191P, G193D and G266S) (Haydon *et al*., 2008). In this work, we set out to gain a better understanding of the molecular mechanisms underlying the effects of these mutations. Our principal aim was to use the drug‐dependent *ftsZ* mutants as tools to investigate the mechanism of action of the benzamides, not only with a view to informing future drug‐development, but also to aid in our understanding of Z‐ring assembly, multiple aspects of which remain poorly understood.

## Results

### Multiple defects accompany benzamide dependence in *S*. *aureus*

**Figure 2:**
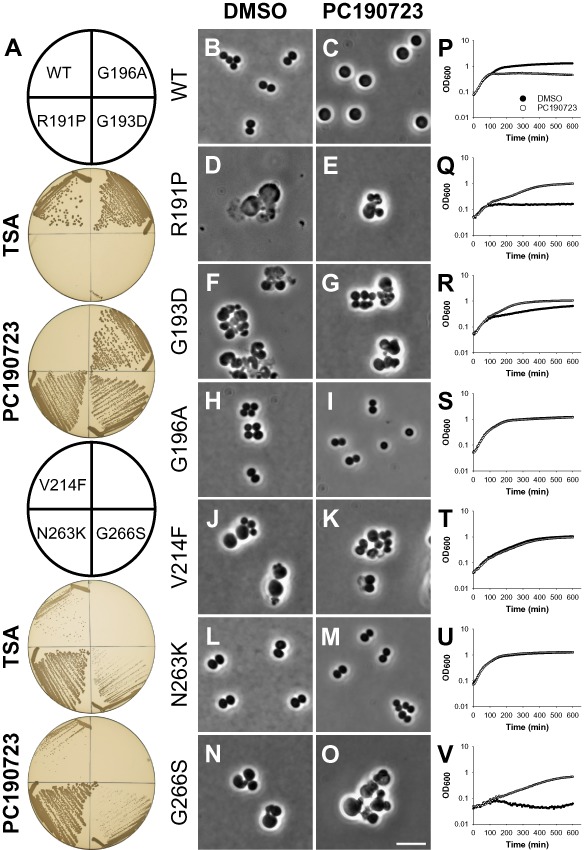
PC190723‐resistant and ‐dependent *S*. *aureus* mutants exhibit multiple growth and morphological defects. A. Growth of *S*. *aureus* mutants on solid media. Strains ATCC 29213 (WT), R191P, G193D, G196A, V214F, N263K and G266S were streaked on tryptic soy agar (TSA) in the absence and presence of PC190723 (8 μg ml^−1^), as indicated. Plates were photographed after incubation at 37°C for 18 h. B–V. Morphology and growth of *S*. *aureus* mutants in liquid media. Exponentially growing cells of strains ATCC 29213 (WT), R191P, G193D, G196A, V214F, N263K and G266S were grown in tryptic soy broth (TSB) at 37°C and observed following growth for 2 h in the presence of either DMSO (1% v/v) (B, D, F, H, J, L, N) or PC190723 (8 μg ml^−1^) (C, E, G, I, K, M, O), as indicated. (P‐V) Strains ATCC 29213 (WT), R191P, G193D, G196A, V214F, N263K and G266S were grown in TSB in the presence of either DMSO (1% v/v) or PC190723 (8 μg ml^−1^), as indicated. The optical density at 600 nm was measured at 6 min intervals and the mean of six replicates plotted against time. Pre‐cultures of WT and resistant strains (G196A, V214F and N263K) were grown in TSB without compound. Pre‐cultures of dependent strains (R191P, G193D and G266S) were grown in TSB containing PC190723 (8 μg ml^−1^).

To investigate the growth and morphology of the *S. aureus ftsZ* mutant strains, they were each grown in the absence and presence of PC, on solid and in liquid media (Fig. 2). As expected, WT *S. aureus* was unable to grow on plates in the presence of PC (Fig. 2A) and in liquid media cells grown with PC rapidly doubled in size [average diameter of 2.0 ± 0.2 μm (*n* = 62) treated versus 1.1 ± 0.1 μm (*n* = 224) untreated] to form enlarged ‘balloons’ that ceased to grow (Fig. 2B, C and P), characteristic of a block in cell division (Pinho and Errington, 2003). In contrast, the PC‐resistant strains producing either FtsZ G196A or FtsZ N263K both exhibited robust growth regardless of the presence or absence of PC. Cells of these strains appeared morphologically normal under either condition and grew at rates indistinguishable from that of the WT parent grown without PC (Fig. 2H, I and S and Fig. 2L, M and U). Both strains did, however, exhibit a minor cell size defect (∼10–20% larger than WT) that was reversed by growth in the presence of PC (compare Fig. 2H versus I and L versus M). The remaining *ftsZ* mutants all exhibited severe growth and morphological phenotypes (summarised in Fig. 2 and Table 1.) that in all cases involved the generation of a wide range of large abnormal cell types undergoing multiple apparently uncoordinated division events. These defects could not be rescued with increased PC concentrations (≤ 32 μg ml^−1^; not shown).

**Table 1:** Summary of *fts* *Z* mutant phenotypes in *S*. *aureus*

Strain	Growth on solid media			Colony size	Cell morphology in liquid media		*B. subtilis* phenotype
	TSA	+ PC	+3MBA		DMSO	+ PC	
WT	Yes	No	No	Normal	Normal	Balloons	–
FtsZ R191P	No	Yes	No	Small	Balloons, lysis	Abnormal	Lethal
FtsZ G193D	No	Yes	Yes	Small	Abnormal, lysis	Abnormal	Lethal
FtsZ G196A	Yes	Yes	No	Normal	Normal	Normal	PC/8J^R,b^
FtsZ V214F	Yes	Yes	Yes	Small	Abnormal	Abnormal	N.D.
FtsZ N263K	Yes	Yes	Yes	Normal	Normal	Normal	Benzamide^R^
FtsZ G266S	Yes^a^	Yes	Yes	Small	Abnormal	Abnormal	3‐MBA^R^

a For reasons that we do not understand, strain G266S grew equally well in the absence and presence of PC on solid media but exhibited a dependent phenotype in liquid media.

This work (G196S) and Haydon *et al*. (2008) (G196A).

N.D., not determined; R, resistant.

To study the resistant and dependent mutants in more detail we attempted to introduce the various substitutions into *Bacillus subtilis* FtsZ using site‐directed mutagenesis (see *Experimental procedures*). Using the same method (Haydon *et al*., 2008) previously showed that a *B. subtilis* mutant producing the FtsZ G196A substitution was PC resistant. Additionally, during the course of this work, a spontaneously arising mutant encoding FtsZ G196S was isolated that behaves similarly (results below). Introduction of the N263K substitution rendered *B. subtilis* resistant to PC and 3‐MBA and had no discernible morphological defect, in agreement with the phenotype of the corresponding mutant in *S. aureus* (Fig. S1). Curiously, and in contrast to its effects in *S. aureus*, the G266S substitution rendered *B. subtili*s resistant to 3‐MBA but not PC (not shown). Multiple attempts to introduce the R191P and G193D substitutions were unsuccessful regardless of the presence or absence of the various compounds, suggesting that under the tested conditions, these mutations are lethal in *B. subtilis*.

### FtsZ G196 substitutions are PC‐resistant but 3‐MBA sensitive

**Figure 3:**
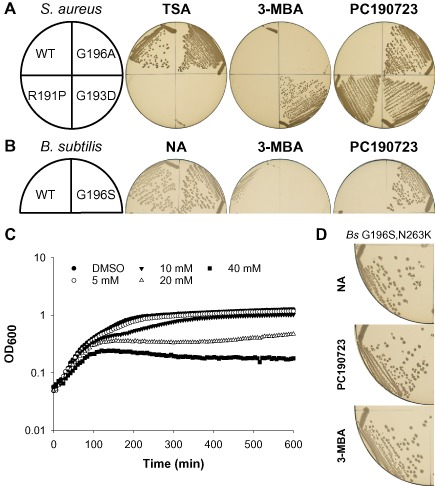
FtsZ G196 substitutions are PC190723 resistant but 3‐MBA sensitive. A. *S*. *aureus* strains ATCC 29213 (WT), R191P, G193D and G196A, and (B) *B*. *subtilis* strains 168 (WT) and DWA23 (FtsZ G196S) were streaked on TSA and nutrient agar (NA) respectively, in the absence and presence of either 3‐MBA (10 mM) or PC190723 (8 μg ml^−1^), as indicated. Note that the same TSA and PC plates are also shown in Fig. 2A. C. *S*. *aureus* strain G196A was grown in TSB at 37°C in the presence of either DMSO (1% v/v) or a series of dilutions of 3‐MBA (mM; as indicated). The optical density at 600 nm was measured at 6 min intervals and the mean of six replicates plotted against time. D. Strain DWA27 (*ftsZ cat*[FtsZ G196S, N263K]) was streaked on NA + Cm, in the absence and presence of either PC190723 (8 μg ml^−1^) or 3‐MBA (10 mM), as indicated. Plates were photographed after incubation at 37°C for 18 h.

To investigate the mechanism(s) underlying the drug‐resistant and drug‐dependent *ftsZ* mutations, the ability of these strains to grow in the presence of the original hit compound 3‐MBA was also examined. *S. aureus* strains producing the FtsZ variants G193D, V214F, N263K and G266S were all able to grow in the presence of 3‐MBA, whereas the WT parent was not (Fig. 3A and Fig. S2). Unexpectedly, however, growth of the *S. aureus* strains bearing the PC‐resistant substitution G196A or the PC‐dependent substitution R191P did not occur in the presence of 3‐MBA (Fig. 3A). Due to the gross morphological defects exhibited by strain R191P and the availability of an equivalent G196 substitution in *B. subtilis*, it was not studied further.

**Figure 4:**
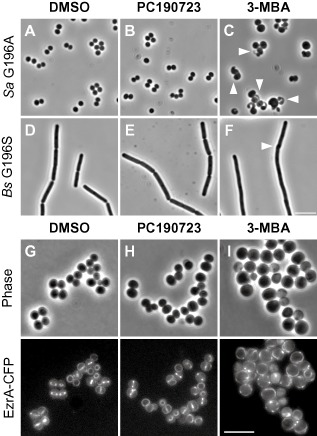
3‐MBA sensitive mutants are specifically affected in cell division. (A–F) Morphology of *B*. *subtilis* and *S*. *aureus* mutants sensitive to 3‐MBA. (A–C) *S*. *aureus* strain G196A and (D–F) *B*. *subtilis* strain DWA23 (FtsZ G196S) were grown at 37°C in TSB and LB respectively. Cells were examined by phase‐contrast microscopy following growth for 2 h in the presence of DMSO (1% v/v) (A and D), PC190723 (8 μg ml^−1^) (B and E) and 3‐MBA (10 mM) (C and F), as indicated. Arrowheads highlight unusual cell division events. (G–I) Localisation of EzrA‐CFP in a *S*. *aureus* FtsZ G196A background. Cells of strain DWA56 (EzrA‐CFP, FtsZ G196A) were grown at 30°C in TSB containing DMSO (1% v/v) (G), PC190723 (4 μg ml^−1^) (H) and 3‐MBA (20 mM) (I), as indicated. Scale bars = 5 μm.

As described above, the growth of *S. aureus* strain G196A is similar to that of the WT parent and is unaffected by PC. In contrast, the strain exhibited a dose‐dependent response to 3‐MBA (Fig. 3C). Indeed, following growth in the presence of 3‐MBA, cells of strain G196A were noticeably larger than those of either the untreated or PC‐treated controls (compare Fig. 4C with A and B) and showed evidence of multiple abnormal division events (Fig. 4C). Importantly, the *B. subtilis* G196S strain behaved similarly. It was unable to grow on plates containing 3‐MBA (Fig. 3B) and formed filaments that occasionally contained unusual helical division events (compare Fig. 4F with D and E), similar to those previously observed in WT cells treated with 3‐MBA (Adams *et al*., 2011).

To examine the effect of 3‐MBA on FtsZ G196A in more detail, the substitution was re‐isolated in a *S. aureus* strain producing EzrA‐CFP, an early‐assembling division protein that serves as a convenient marker for Z‐ring assembly (Pereira *et al*., 2010; Jorge *et al*., 2011; Steele *et al*., 2011). As expected, given its PC‐resistant phenotype, the localisation of EzrA was unaffected by growth in the presence of PC (compare Fig. 4G and H). However, as additional evidence of a direct effect on cell division, the localisation of EzrA was clearly perturbed in cells treated with 3‐MBA (Fig. 4I). In contrast to the discrete rings or bands present in the controls, EzrA localised as a variety of aberrant structures, and often formed intense foci or else, was dispersed in the cell membrane (compare Fig. 4I with G and H).

Given the location of G196 (Fig. 1A), the simplest explanation for the 3‐MBA sensitivity of these strains is that substitutions at this residue are able to occlude the binding of more complex derivatives with large extensions off the benzamide, such as PC, but not smaller derivatives such as 3‐MBA. In contrast, bulky substitutions at N263, which sits at the base of the compound‐binding site (Fig. 1A), would block the binding of both compounds, in agreement with the benzamide‐resistant phenotypes observed for the N263K substitutions in *S. aureus* and *B. subtilis*. Consequently, we predicted that combining the two substitutions would rescue 3‐MBA sensitivity. Indeed, consistent with this hypothesis a *B. subtilis* strain engineered to express FtsZ G196S, N263K is completely resistant to both PC190723 and 3‐MBA (Fig. 3D). Taken together with the cell biology data, these results indicate that the FtsZ G196A/S strains are sensitive to 3‐MBA as it can still bind in the same site and rules out the possibilities that it acts via an off‐target effect, binds to another site on FtsZ, or else that these mutants are resistant to PC via a mechanism distinct from steric occlusion.

### A novel benzamide‐dependent strain of *B*. *subtilis*

**Figure 5:**
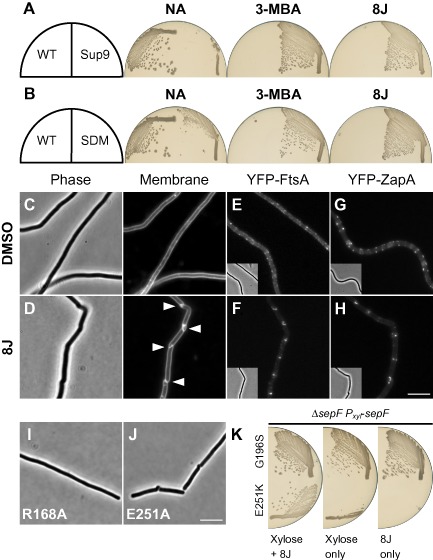
A benzamide‐dependent strain of *B*. *subtilis*. A. Strain DWA454 is benzamide dependent. Strains 168 (WT) and DWA454 (*ftsZ* sup9[FtsZ E251K]) were streaked on NA in the absence and presence of either 3‐MBA (10 mM) or 8J (2 μg ml^−1^), as indicated. B. The re‐engineered *ftsZ* sup9 mutation is sufficient for benzamide dependence. Strains DWA458 (*ftsZ cat*[FtsZ WT]) and DWA455 (*ftsZ cat*[FtsZ E251K]) (Site‐directed‐mutant; SDM) were streaked on NA + Cm in the absence and presence of either 3‐MBA (10 mM) or 8J (2 μg ml^−1^), as indicated. Plates were photographed after incubation at 37°C for 18 h. C and D. Cell division is benzamide dependent. Exponentially growing cells of strain DWA454 were examined following growth in the absence (C) and presence (D) of 8J (8 μg ml^−1^). Arrowheads highlight unusual cell division events. Cell membranes were stained with FM5‐95. E–H. Localisation of early‐assembling division proteins in the benzamide‐dependent strain background. Exponentially growing cells of strains DWA638 (FtsZ E251K, YFP‐FtsA) and DWA637 (FtsZ E251K, YFP‐ZapA) were observed following growth in the absence (E and G) and presence (F and H) of 8J (8 μg ml^−1^). I and J. Cell division defects associated with mutations at R168 and E251. Strains DWA410 (*ftsZ cat*[FtsZ R168A]) and DWA412 (*ftsZ cat*[FtsZ E251A]) were observed after growth overnight at 37°C on NA + Cm. Scale bars = 5 μm. K. *sep* *F* is synthetic lethal with *ftsZ* sup9. Strains DWA460 (Δ*sepF*, *P_xyl_‐sep* *F*, FtsZ G196S) and DWA461 (Δ*sepF*, *P_xyl_‐sepF*, FtsZ E251K) were streaked on plates containing 0.5% w/v xylose, 8J (2 μg ml^−1^) and both, as indicated. Plates were photographed after incubation at 37°C for 18 h.

The various defects of the PC‐dependent *S. aureus* strains precluded a more detailed analysis. Therefore, to facilitate further study of this class of mutation, a screen was conducted for spontaneous benzamide‐dependent mutants of *B. subtilis* (see *Experimental procedures*). One candidate, designated sup9, was identified that was able to grow only in the presence of 8J (≥ 0.2 μg ml^−1^) or 3‐MBA (≥ 10 mM) (Fig. 5A). PC was equally able to support growth (not shown). Thus, the strain is benzamide‐dependent.

To test whether the dependent phenotype was due to an effect on division, sup9 was cultured in liquid media. Indeed, in the absence of any additions, cells formed long filaments (Table 2), characteristic of a cell division block (Fig. 5C). In contrast, cells grown in the presence of 8J were able to divide regularly (Fig. 5D), although they were on average twofold longer than those of the untreated WT parent (Table 2). Unexpectedly, however, at all tested concentrations of 8J, these division events frequently (89%; 224/251 cells) occurred in a helical manner (Fig. 5D) and appeared morphologically similar to those observed in WT cells treated with sub‐inhibitory concentrations of 8J (Adams *et al*., 2011). Growth of the strain in the presence of 8J was robust although clearly impaired as compared with the untreated WT control (Fig. S3A and B). This may reflect the tendency of the strain to form long chains of cells, which possibly as a result of the unusual mode of division, often appeared to be unstable and prone to lysis (Fig. S4). Curiously, although in the absence of 8J *c.* 90% cells formed filaments, growth was equally robust and did not show evidence of lysis, indicating that the cells likely retain the ability to divide, albeit only occasionally (Fig. S3B and Table 2). The observation that *c.* 10% of the cells grown without 8J were ≤ 10 μm is consistent with this idea. The discrepancy between these results and the inability of the strain to grow on solid media in the absence of 8J may result from the reduced stability of long filaments on agar surfaces, which may not allow sufficient time for the rare division events to occur.

**Table 2:** Cell lengths of *B*. *subtilis* strains producing *fts* *Z* mutants

Strain	FtsZ^a^	Condition^b^	Mean cell length (μm)	Standard deviation (μm)	Fold change versus WT	Filaments^c^ (%)	N^o^ of cells
168	WT	N/A	2.8	0.7	–	0	231
DWA454	E251K	DMSO	27.5	16.8	9.8	90.2	132
		+8J	5.7	1.9	2.0	2.9	238
DWA456	I228T, E251K	DMSO	6.5	2.6	2.3	9.1	263
		+8J	5.1	2.6	1.8	4.5	242
DWA458	WT Cm^R^	+ Cm	2.7	0.7	–	0	240
DWA401	I228L	+ Cm	3.5	1.3	1.3	0.9	225
DWA402	I228V	+ Cm	3.6	1.6	1.3	1.2	256
DWA414	I228V, E251K	+ Cm	4.5	1.7	1.7	0.4	266
DWA419	I228L, E251K	+ Cm	3.5	1.2	1.3	0	332

a FtsZ was produced at comparable levels in all strains tested (Fig. S7).

b Cells were grown to exponential phase in LB at 37°C in the presence of no additions (N/A), 1% v/v DMSO, 8 μg ml^−1^ 8J or chloramphenicol (Cm), as indicated. Cell lengths were measured using FM5‐95 stained cells.

c Filament defined as cell length > 10 μm.

To test at which step the division block occurs, the sup9 mutation was moved into strains producing YFP‐fusions to the early‐assembling division proteins FtsA and ZapA. In cells grown with 8J, both proteins localised predominantly at the division sites, often as a short helical structure (Fig. 5F and H), consistent with the unusual geometry of the division event. Conversely, when the compound was removed, FtsA and ZapA formed discrete foci that were scattered along the cell membrane of the filament (Fig. 5E and G), indicating that the division block occurs at the level of Z‐ring assembly. Interestingly, the distributions of some of these foci appear compatible with short helical structures, suggesting that FtsZ may be blocked at the transition from polymer to Z‐ring (see *Discussion*).

### A substitution at the FtsZ inter‐domain interface is responsible for benzamide dependence

DNA sequencing revealed that sup9 contained a single point mutation in *ftsZ*, resulting in the substitution of a glutamic acid for a lysine at residue 251. To rule out the possibility that mutations in other genes were required, the mutation was introduced into an otherwise WT strain. Importantly, the resulting strain, carrying only the re‐engineered *ftsZ* mutation, was benzamide dependent, demonstrating that this mutation alone is both necessary and sufficient for the phenotype (Fig. 5B).

**Figure 6:**
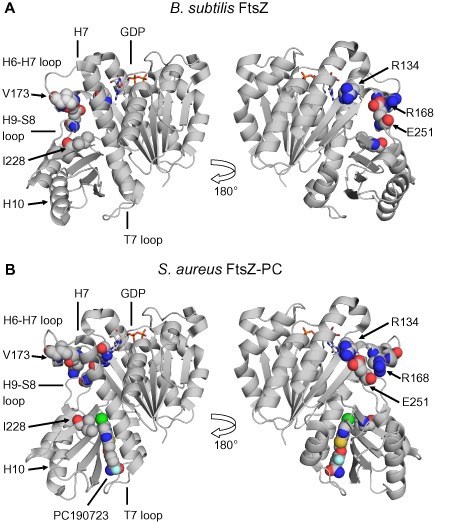
Structural context of FtsZ substitutions. Cartoon representations of (A) *B. subtilis* FtsZ‐GDP (PDB 2RHL) and (B) *S*. *aureus* FtsZ‐PC190723‐GDP (PDB 3VOB) complexes. Images were prepared using PyMOL after superimposing the N‐terminal domains (residues 13–173) and are shown in the same orientation. The right‐hand panels are rotated 180°, as indicated. PC and residues altered by *ftsZ* mutations described in the text are shown as spheres and highlighted features are coloured according to chemical element.

E251 does not form part of the benzamide‐binding site. It is located at the rear of the C‐terminal domain, on a surface exposed loop that sits at the interface between the N and C‐terminal domains (Fig. 6A). In the available crystal structures of *B. subtilis* FtsZ, the residue closest to E251 is generally arginine 168 (Fig. 6A). As the carboxylate of E251 and the guanidinium of R168 are typically within a few angstroms of each other (e.g. 2.8–3.4 Å in PDB 2VXY), they have the potential to form a salt‐bridge. Furthermore, a species‐wide alignment of 500 non‐redundant FtsZ sequences indicated that the opposing charges at these positions are well conserved (168 – K, 49%; R, 28% and 251 – E, 44%; D, 44%). To test whether these residues are important for normal FtsZ function, we created new mutations that should disrupt any potential interaction. Replacement of E251 with glutamine, which is similar in size but uncharged, rendered cells 3‐MBA dependent (Fig. S5). In contrast, mutants encoding alanine substitutions at E251 and R168 were only obtained at low frequency and formed small colonies. Neither of the strains was benzamide resistant and both had a division defect. Cells producing FtsZ R168A formed long filaments (Fig. 5I), whereas those producing FtsZ E251A consisted of a mixture of filamentous and shorter cells that contained helical cell divisions (Fig. 5J).

### *fts* *Z* sup9 is synthetic‐lethal with *sep* *F*

To test for additional phenotypes, we attempted to combine the *ftsZ* sup9 mutation with deletions in genes of known FtsZ regulators and Z‐ring accessory proteins, i.e. *ftsA*, *ezrA*, *minD*, *noc*, *sepF*, *ugtP* and *zapA*. The PC/8J‐resistant *ftsZ* mutant (G196S) was used as a negative control and had no additional phenotype when combined with any of the secondary mutations. Strains transformed with *ftsZ* sup9 were benzamide‐dependent and on the whole the double mutant cells utilised the same unusual mode of division. An exception to this was the *ezrA* double mutant, which formed a mixture of elongated cells and short filaments in which division appeared to proceed conventionally. However, Δ*ezrA* cells treated with sub‐inhibitory concentrations of 8J that are sufficient to induce helical cell division in the WT parent, behaved similarly (Fig. S6). This suggests that either EzrA is required for these unusual division events or else its absence is able to suppress them. Nevertheless, as the effect is not specific to the *ftsZ* mutation, it was not studied further.

The second exception was the *sepF* deletion, which could not be combined with the *ftsZ* sup9 mutation. To verify this phenotype, the experiment was repeated in a strain containing a conditional allele of *sepF* under the control of a xylose‐inducible promoter. Indeed, growth of this stain was dependent on the presence of both xylose and 8J, indicating that *ftsZ* sup9 is synthetic‐lethal with *sepF* (Fig. 5K). Interestingly, this effect was not caused indirectly by the unusual mode of division as Δ*sepF* cells treated with sub‐inhibitory concentrations of 8J divide in a similarly unusual manner (not shown).

### Assembly of FtsZ E251K *in vitro* is benzamide‐independent

**Figure 7:**
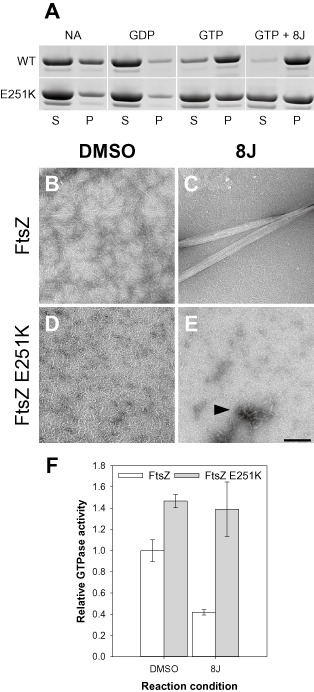
FtsZ E251K assembles *in vitro* in a benzamide‐independent manner. A. Sedimentation assays of FtsZ and FtsZ E251K. Proteins (10 μM) were prepared in polymerisation buffer (50 mM HEPES pH 6.8; 50 mM KCl; 10 mM MgCl_2_) with no additions (NA), or in the presence of GDP (1 mM), GTP (1 mM) and GTP + 8J (1 mM and 20 μg ml^−1^), as indicated. Reactions were incubated at 30°C for 10 min, and assembly was assayed by sedimentation. S, supernatant; P, pellet. B–E. Examination of FtsZ polymers by electron microscopy. FtsZ and FtsZ E251K (10 μM) were assembled in polymerisation buffer in the absence (B and D) and presence (C and E) of 8J (20 μg ml^−1^). Polymerisation was initiated by the addition of GTP (1 mM), and reactions were incubated at 30°C for 10 min before being prepared for electron microscopy. Scale bar = 200 nm. F. FtsZ E251K GTPase activity is unaffected by 8J. FtsZ and FtsZ E251K (10 μM) were prepared in polymerisation buffer in the absence (DMSO only) and presence of 8J (20 μg ml^−1^). Polymerisation was initiated by the addition of GTP (1 mM), and the reactions were incubated at 30°C for 30 min. GTPase activity was determined using the malachite green assay, and for each condition, the mean of three independent experiments was plotted. Error bars represent one standard deviation.

In cells of sup9 grown without 8J, the early assembling division proteins were not uniformly diffuse. This suggested that in the absence of benzamide, FtsZ E251K is able to assemble, albeit non‐productively. To test this hypothesis, the E251K variant was purified. Preliminary biochemical experiments indicated that like the unmodified protein, FtsZ E251K assembles *in vitro* in a GTP‐dependent manner and has comparable levels of GTPase activity (Fig. 7A and F). Similarly, assembly was further stimulated by the addition of 8J (Fig. 7A), although this effect was clearly reduced as compared with the WT protein in which essentially all the protein was recovered in the pellet (see below). However, in contrast to the inhibitory effect observed with the wild‐type FtsZ, the GTPase activity of FtsZ E251K was unaffected by the addition of the 8J (Fig. 7F). To investigate if differences in polymer morphology could be detected, electron microscopy was used to examine the polymerisation reactions. In the absence of the compound both proteins generally formed single‐stranded proto‐filaments *c.* 100–200 nm in length (Fig. 7B and D). FtsZ polymers assembled in the presence of 8J formed large ribbons *c.* 100–200 nm in diameter and up to several microns in length, which contained numerous proto‐filaments, as previously described (Fig. 7C) (Andreu *et al*., 2010). Conversely, these distinctive structures were not observed for FtsZ E251K, and the predominant polymer species was the single stranded proto‐filament (Fig. 7E), though in agreement with the pelleting results, some large multi‐stranded structures were still apparent, albeit infrequently (arrowhead in Fig. 7E).

### An intragenic suppressor of benzamide‐dependence

**Figure 8:**
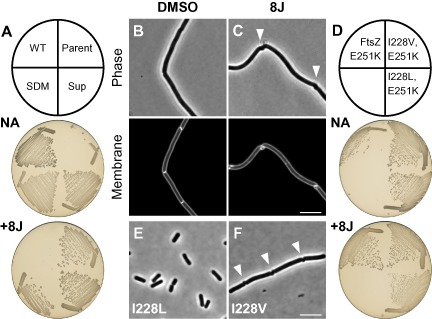
An intragenic suppressor of benzamide dependence. A. Suppressors of benzamide dependence. Strains 168 (WT), DWA454 (*ftsZ* sup9[FtsZ E251K]), DWA456 (*ftsZ* sup9^sup^[FtsZ I228T, E251K]) and DWA33 (*ftsZ cat*[FtsZ I228T, E251K]) (SDM) were streaked on NA in the absence and presence of 8J (2 μg ml^−1^), as indicated. Plates were photographed after incubation for 18 h at 37°C. B and C. DWA456 divides in a benzamide‐independent manner. Exponentially growing cells of strain DWA456 were examined by microscopy following growth in the absence (B) and presence (C) of 8J (2 μg ml^−1^). Arrowheads highlight unusual cell division events. Cell membranes were stained with FM5‐95. Scale bar = 5 μm. D. I228V and I228L also provide benzamide independence. Strains DWA455 (*ftsZ cat*[FtsZ E251K]), DWA414 (*ftsZ cat*[FtsZ I228V, E251K]) and DWA419 (*ftsZ cat*[FtsZ I228L, E251K]) were streaked on NA + Cm in the absence and presence of 8J (2 μg ml^−1^), as indicated. Plates were photographed after incubation for 18 h at 37°C. E and F. Single mutants encoding I228L and I228V. Strains DWA401 (*ftsZ cat*[FtsZ I228L]) (E) and DWA402 (*ftsZ cat*[FtsZ I228V]) (F) were observed after growth overnight at 37°C on NA + Cm. Arrowheads in (F) highlight unusual cell division events. Scale bar = 5 μm.

Suppressors of benzamide‐dependence readily emerged following culture in the absence of benzamide. Notably, these strains were able to grow on plates with or without 8J and grew at similar rates in liquid media (Fig. 8A and Fig. S3C). In the absence of any additions division appeared to proceed relatively normally in most cells (65%, 143/221 cells) (Fig. 8B). However, cells grown in the presence of 8J again predominantly utilised an unusual helical mode of division (91%, 199/218 cells) (Fig. 8C). Under both conditions cells were on average approximately twofold longer than the untreated WT parent (Table 2). DNA sequencing revealed that in these strains the parental *ftsZ* sup9 mutation was now accompanied by a second point mutation encoding the substitution of isoleucine 228 to threonine. Recreation of the double mutant in otherwise WT cells confirmed that this second mutation is both necessary and sufficient for the benzamide‐independent phenotype (Fig. 8A).

Residue I228 is located in the benzamide‐binding site within the hydrophobic cleft between H7 and the C‐terminal domain that accommodates the thiazolopyridine moiety of PC (Fig. 6A and B). In the presence of PC, the side‐chain of I228 is rotated away from this moiety, presumably to avoid a steric clash (Fig. 6B). Interestingly, it was not possible to make an I228T single mutant (not shown). Threonine is smaller than isoleucine and contains a polar side‐chain, which might disrupt this predominantly hydrophobic region. Alternatively, the side‐chain hydroxyl could introduce a stabilising interaction with the amide of glutamine 192, which sits opposite. In an attempt to distinguish between these possibilities, we tested whether other substitutions (i.e. alanine, glycine, leucine and valine) were tolerated and whether they could suppress the benzamide‐dependent phenotype. The larger and hydrophobic I228V and I228L substitutions were both able to suppress the benzamide‐dependency of E251K and the double mutants generally behaved similarly to the I228T substitution (Fig. 8D). Interestingly, the conservative change I228L exhibited the shortest cell length of the three double mutants tested (Table 2). Individually, both substitutions were also tolerated, though neither provided any benzamide resistance (not shown). Cells producing FtsZ I228L and I228V both had a minor cell length defect and were *c.* 30% longer than the WT parent (Table 2). Additionally, whereas cells producing I228L divided normally (Fig. 8E), 15% cells producing FtsZ I228V (86/572 cells) contained helical division events (Fig. 8F). In contrast to these results, single or double mutants encoding I228A or I228G could not be obtained under any of the conditions tested.

## Discussion

### Potential clinical relevance of *S*. *aureus fts* *Z* mutants

The benzamides represent the best‐studied FtsZ inhibitors and are promising leads for a new class of antibiotics. Nevertheless, the frequency of resistance to PC poses a substantial challenge to future drug development (Tan *et al*., 2012; Stokes *et al*., 2013). However, of the six spontaneously arising *S. aureus* mutants studied here, only two (G196A and N263K) exhibited morphologies and growth rates similar to that of the untreated WT parent. Similar results were obtained with the equivalent substitutions in *B. subtilis*. The remaining *S. aureus* mutants (R191P, G193D, V214F and G266S) all exhibited a variety of severe growth and morphological defects. Moreover, it was not possible to generate the PC‐dependent R191P or G193D FtsZ variants in *B. subtilis*, which likely reflects the inability of a rod‐shaped bacterium to accommodate the diverse division geometries that they generate, as easily as in a spherical cell.

As noted by Stokes *et al*. (2013), ‘Understanding the potential for the emergence of resistance, as well as the viability or fitness of spontaneous inhibitor‐resistant mutants, is important in a single‐enzyme‐targeted antibacterial drug discovery program’. Thus, the results described here predict that some of these mutants would be unlikely to proliferate in the clinic, as in their compromised state, the mutations would likely be unstable and/or the cells would exhibit attenuated virulence. Indeed, in support of this hypothesis, the G193D FtsZ variant is sufficient to impair the virulence of Methicillin‐resistant *Staphylococcus aureus* (MRSA) in a murine model of infection, whereas G196S and N263Y variants were unaffected (Tan *et al*., 2012). The G193D variant is also sufficient to re‐sensitise MRSA to imipenem (Tan *et al*., 2012). The multiple unusual and apparently uncoordinated division events exhibited by this strain may explain this effect as PBP2, the target of imipenem, will likely be diluted at these sites or alternatively the unusual divisions may lead to a weakened cell wall.

PC‐resistant mutants isolated in MRSA commonly encode FtsZ substitutions at residues G196, G193 and N263 (Tan *et al*., 2012; Kaul *et al*., 2015). However, the most frequently occurring substitutions are at G196 (Haydon *et al*., 2008) and account for around 70–75% of all isolates with G196S being the most common substitution (Tan *et al*., 2012; Kaul *et al*., 2015). The finding here that both *S. aureus* G196A and *B. subtilis* G196S strains are sensitive to the small benzamide 3‐MBA supports the crystallographic data showing that even small changes at this residue would block the binding of more advanced benzamides like PC and 8J (Matsui *et al*., 2012; Tan *et al*., 2012). Likewise, the demonstration that 3‐MBA‐sensitivity is rescued by introducing the N263K substitution is consistent with a model whereby these substitutions act by differentially occluding benzamide binding. Additionally, these results [and others (Haydon *et al*., 2008)] validate the benzamide‐binding site in *B. subtilis* FtsZ and thus directly refute the recent suggestion that PC does not bind to the same site as in *S. aureus* (Miguel *et al*., 2015). Importantly, these results also suggest that benzamides with alternative extensions can be designed that would not be susceptible to occlusion at G196. Indeed, benzamides with improved pharmacokinetic properties that exhibit superior *in vitro* and *in vivo* antibacterial efficacy have recently been reported (Stokes *et al*., 2013; 2014). Significantly, these compounds retain potent on‐target activity against the G196A substitution (Stokes *et al*., 2013). Substitutions at N263, however, may prove more challenging to overcome as the residue sits directly adjacent to the benzamide and is thus not amenable to optimisation. Nevertheless, it is curious that although *S. aureus* strains bearing N263K substitutions have no overt additional phenotype, they are isolated in MRSA at the same low frequency (*c.* 10–15%) as G193D (Tan *et al*., 2012; Kaul *et al*., 2015) and were not isolated at higher concentrations of the improved benzamides (Stokes *et al*., 2013).

### Origin and possible mechanisms of benzamide‐dependence

The benzamides normally act as bactericidal compounds by blocking division at the level of Z‐ring assembly (Haydon *et al*., 2008; Adams *et al*., 2011). In contrast, we have isolated a novel benzamide‐dependent *ftsZ* mutation in *B. subtilis* encoding the substitution E251K. In the absence of the benzamides, cells of this strain fail to divide and form long filaments due to a block at the level of Z‐ring assembly. In the presence of benzamides division is restored. However, these division events are unusual and occur in a helical manner. As the benzamides are known to enhance FtsZ assembly (Andreu *et al*., 2010; Adams *et al*., 2011), the simplest explanation would be that they are required to facilitate polymer assembly. However, the data argue against this possibility. First, in cells grown without additions, early‐assembling division proteins are recruited to multiple foci and loose helical structures scattered throughout the cell, indicative of non‐productive FtsZ assembly. Second, purified FtsZ E251K is able to assemble *in vitro* in a GTP‐dependent manner independently of the benzamides to levels comparable with FtsZ. Nevertheless, as it is unable to support Z‐ring assembly, it presumably has some structural defect beyond the level of basic proto‐filament assembly that traps the protein at the transition between a polymer and a Z‐ring. Third, in contrast to unmodified FtsZ, FtsZ E251K did not form large multi‐stranded polymer bundles in the presence of 8J, strongly suggesting that they do not rescue division by promoting polymer assembly *per se*.

How then might this mutation act? Residue E251 is located outside of the benzamide‐binding site and sits on the H9‐S8 loop at the surface of the C‐terminal domain (Fig. 6A). In most FtsZ crystal structures, E251 is proximal to R168, which sits on H6 in the N‐terminal domain (Fig. 6A), at an appropriate distance to form a salt‐bridge. Single mutations designed to disrupt this interaction, i.e. E251A and R168A both had a division defect indicating that these residues are important for normal FtsZ function and supporting the idea that they interact. Importantly, the larger, positively charged side‐chain of lysine is not compatible with such an interaction. Consequently, the H9‐S8 loop and H6 would likely have increased flexibility leading to a loss of rigidity in these domains. As neither E251A nor R168A was benzamide‐dependent, electrostatic repulsion of R168 by E251K is likely the source of benzamide‐dependence and might act to further perturb the orientation of the H6‐H7 loop. Previously, Ohashi *et al*. (1999) reported that FtsZ V173F was 3‐MBA‐dependent. Notably, V173 sits on the same helix as R168, suggesting that the effects of the two substitutions might share a common mechanism (Fig. 6).

The benzamide‐dependent phenotype of this strain provides evidence that benzamide binding may counteract or otherwise stabilise these distortions at the inter‐domain interface. Strikingly, in crystal structures of the *S. aureus* FtsZ‐GDP‐PC complex, residue E251 is reoriented away from R168 (e.g. 5.8–7.4 Å in PDB 3VOB) and instead contacts residue R134 (Fig. 6B). As R134 is separated from the H6‐H7 loop, the repulsive effects of E251K may be minimised. Nevertheless, the ability of the benzamides to restore Z‐ring assembly is only partial as cells divide using unusual helical divisions, indicating that Z‐ring assembly remains defective at one or more levels. Indeed, the observation that the normally redundant role of SepF, which is thought to form arcs at the nascent septum that link FtsZ polymers to the membrane and align them for Z‐ring assembly (Duman *et al*., 2013), becomes essential in this strain supports the idea that the polymer morphology remains defective.

Overall, the data lead to two possible models. First, as the *B. subtilis* structure is thought to represent the ‘curved’ or relaxed form of FtsZ and the *S. aureus* structures the ‘straight’ or active form (Matsui *et al*., 2012; 2014), switching between the interactions of E251 and R168 may regulate the FtsZ assembly cycle. In this model, repulsion of R168 on the H6‐H7 loop by E251K might push the longitudinal interface towards, or even block it in, the curved state. Indeed, this loop also contacts H10 of the subunit above at the polymerisation interface and could therefore transmit the local perturbation throughout the polymer (Fig. 6). Thus, benzamide binding would serve to tip the balance back towards the straight or active form. Consequently, the helical division phenotype of the strain may result from a balance between the two states or else a weakened effect of the benzamides caused by the altered starting state of the mutant polymer. This might explain how the mutant is able to function with a benzamide in the inter‐domain cleft, which likely opens and closes during the normal FtsZ assembly cycle.

Alternatively, given that E251 and R168 are located towards the side of the molecule, it is possible that the mutant may be defective in lateral interactions, i.e. the ability to interact with a molecule in an adjacent proto‐filament. In this second model, the E251K substitution, either directly or via the repulsion the H6‐H7 loop, causes a defect in lateral interactions that is restored by benzamide binding. The synthetic‐lethal effect with *sepF* could also support this idea. However, as described above, the *in vitro* data do not support this model. Moreover, under more physiological conditions, the benzamides promote the formation of highly curved single‐stranded FtsZ proto‐filaments, strongly suggesting that they act primarily at the longitudinal interface (Andreu *et al*., 2010; Adams *et al*., 2011). Similarly, unlike the well‐characterised longitudinal interface, the existence of lateral interactions between FtsZ polymers remains unclear, and it has been suggested that any contacts may either be transient or else mediated by another protein(s) (Erickson *et al*., 2010; Szwedziak *et al*., 2014). Indeed, the link between the bundling of proto‐filaments *in vitro* and the function of the Z‐ring *in vivo* is also uncertain as some FtsZ variants that form bundles *in vitro* fail to function *in vivo* and vice versa (Lu *et al*., 2001; Koppelman *et al*., 2004; Buske and Levin, 2012). Further biochemical work will clearly be necessary to differentiate between these two models, e.g. conditions that favour lateral interactions and ultimately structural studies may be needed. Nevertheless, understanding the exact source of the defect will be challenging, as *in vitro* FtsZ E251K polymers appeared largely unperturbed. Another complicating factor is that the precise nature of the polymers required for Z‐ring assembly remains unknown.

An intragenic suppressor of E251K, encoding I228T, was isolated that allowed division regardless of the presence or absence of benzamide. Substitutions to other branched chain amino acids, i.e. I228L and I228V behaved similarly and could also be introduced in isolation. However, substitutions encoding smaller amino acids, i.e. I228A and I228G could not be introduced, indicating that the size of the side‐chain is important. Indeed, isoleucine and leucine differ only in the arrangement of the side‐chains so even subtle changes may be sufficient to restore function. I228T may act via a distinct mechanism as it was not possible to make the single mutant. I228 sits at the top of the benzamide‐binding site (Fig. 6) so these substitutions presumably mimic some aspect of compound binding and perhaps act to wedge the cleft in the open state. However, as all the suppressors allowed apparently normal cell division in the absence of benzamide, these effects may be reversible, especially as compared with the effects of benzamide binding. An important next step will be to test whether substitutions at I228 affect either FtsZ assembly or proto‐filament morphology *in vitro* and may help to clarify the mechanism(s) of both benzamide‐dependence and of suppression.

Overall, these results provide genetic evidence that benzamides can restrict or otherwise counteract conformational changes in FtsZ and are consistent with the suggestion that these compounds act by blocking the switch from the active (cleft‐open) to inactive (cleft‐closed) states that probably occurs at some point post GTP hydrolysis (Matsui *et al*., 2012; 2014; Ramírez‐Aportela *et al*., 2014). The conservation of residues equivalent to *B. subtilis* R168 and E251 also suggests a potentially important role for this region across bacterial species. Notably, several other substitutions affecting Z‐ring assembly cluster at this region and were sometimes reported to form unusual helical Z‐rings (Wang *et al*., 2001; Stricker and Erickson, 2003; Feucht and Errington, 2005; Haeusser *et al*., 2015). Moreover, previous work suggests that this region may function as a hinge or pivot‐point (Martín‐Galiano *et al*., 2010; Li *et al*., 2013) and recent work in *E. coli* showed that modulation of this region might be exploited by the division‐site selection protein SlmA (Du and Lutkenhaus, 2014). Interestingly, an unrelated class of compounds has been predicted to bind in proximity to this region and appears to act similarly to the benzamides (Kaul *et al*., 2012), reinforcing the idea that this switch may be a useful drug‐target.

## Experimental procedures

### Bacterial strains and plasmids

The bacterial strains and plasmids used in this study are listed in Supplementary Tables S1 and S2 along with the details of their construction.

### General methods

*Bacillus subtilis* cells were made competent for transformation as previously described (Hamoen *et al*., 2002). DNA manipulations and *E. coli* transformations were carried out using standard methods (Sambrook *et al*., 1989), and all constructs were verified by DNA sequencing. Solid medium used for growing bacterial strains was nutrient agar (Oxoid), and liquid medium was Luria–Bertani broth (LB). Solid medium used for growing *S. aureus* was tryptic soy agar (TSA; Bacto), and liquid medium was tryptic soy broth (TSB). Chloramphenicol (Cm; 5 μg ml^−1^), erythromycin (1 μg ml^−1^), spectinomycin (50 μg ml^−1^) and tetracycline (10 μg ml^−1^) were used for selection in *B. subtilis*, as required. For selection in *S. aureus*, the erythromycin concentration was increased to 10 µg ml^−1^, as required. To drive expression from inducible promoters, IPTG (0.2 mM) and xylose (0.5% w/v) were added, as needed. Compounds PC190723 and 8J were supplied by Prolysis. 3‐methoxybenzamide was purchased from Sigma‐Aldrich. Compounds were dissolved in DMSO to create stock solutions and aliquots stored at −20°C.

### Site‐directed mutagenesis of *B*. *subtilis fts* *Z*

*ftsZ* mutations were introduced into the chromosome of *B. subtilis* using derivatives of pSG1928, as previously described (Feucht and Errington, 2005). Briefly, pSG1928 contains a fragment of *ftsZ* that lacks the ATG start codon. Plasmid integration via single cross‐over results in a copy of *ftsZ* under its native promoter linked to a chloramphenicol resistance cassette, followed downstream by the integrated plasmid and a second, truncated copy of *ftsZ* that lacks promoter, ribosome‐binding site and start codon sequences. Site‐directed mutagenesis was used to introduce the desired mutation(s) into pSG1928 using appropriate mutagenic primers (Supplementary Table S3) and PFU Turbo polymerase (Agilent Technologies). The presence of mutations within the constructs and the resulting strains was verified by DNA sequencing.

### Screen for PC190723‐resistant mutants in *S*. *aureus*

Two independent cultures of strain RNpEzrACFP were grown to late exponential phase in TSB at 37°C, spread on plates containing 8 μg ml^−1^ PC and incubated at 37°C for 48 h. Resistant candidates were identified by re‐streaking in the absence and presence of PC, and their identities were confirmed by DNA sequencing.

### Screen for benzamide‐dependent mutants in *B*. *subtilis*

Due to a more limited supply of PC, the closely related benzamide 8J was used to screen for benzamide dependence. Two independent cultures of the wild‐type strain 168 were grown to late exponential phase in LB at 37°C. Aliquots of 100 and 200 μl of each culture were spread on plates containing 8 μg ml^−1^ 8J and incubated at 37°C for 48 h. To re‐screen for compound dependence, the obtained colonies were picked and re‐streaked in the absence and presence of 8J.

### Microscopy

Cells were mounted on microscope slides covered with a thin agarose pad (1.2% w/v in dH_2_O) and were observed using a Zeiss Axiovert 200 M microscope (Carl Zeiss AG, Oberkochen, Germany) attached to a Sony Cool‐Snap HQ cooled CCD camera (Roper Scientific). Cells producing fluorescent protein fusions were grown at 30°C. Cell membranes were stained with either FM5‐95 (4 μg ml^−1^) or Nile‐Red (0.5 μg ml^−1^). Images were prepared for publication using ImageJ (http://rsb.info.nih.gov/ij) and Adobe Photoshop CC.

### Protein purification and FtsZ assembly assays

Untagged FtsZ and FtsZ E251K were purified by ammonium sulphate precipitation and anion‐exchange chromatography as previously described (Adams *et al*., 2011). FtsZ sedimentation assays, negative stain electron microscopy and GTPase activity measurements were done exactly as described in Adams *et al*. (2011) except that the buffer used was 50 mM HEPES pH 6.8; 50 mM KCl; 10 mM MgCl_2_.

## Conflict of interest

JE was a Director and Shareholder in Prolysis Ltd, where the benzamide inhibitors were developed.

## Supporting information

Supporting information
