# Supplementary material for: A benzamide‐dependent fts Z mutant reveals residues crucial for Z‐ring assembly
Source: Mol Microbiol. 2015 Dec 22;99(6):1028–42. doi: 10.1111/mmi.13286 (PMC4832351; doi:10.1111/mmi.13286)
Supplement: Supplementary file 1 — Supporting information [file MMI-99-1028-s001.pdf]

Supplementary information for:

A benzamide-dependent *ftsZ* mutant reveals residues crucial for Z-ring assembly

David William Adams†*, Ling Juan Wu & Jeff Errington*

Centre for Bacterial Cell Biology, Baddiley-Clark Building, Medical School, Newcastle University, Richardson Road, Newcastle upon Tyne, NE2 4AX, United Kingdom

†Present address: Laboratory of Molecular Microbiology, Global Health Institute, School of Life Sciences, Ecole Polytechnique Fédérale de Lausanne, Lausanne, CH-1015, Switzerland.

*Corresponding authors

Email: jeff.errington@ncl.ac.uk; Telephone: +44 (0) 191 208 3235; Fax: +44 (0) 191 208 3205

Email: david.adams@epfl.ch; Telephone: +41 (0) 21 693 0682

Supplementary Figures S1-7

Supplementary Tables S1-3

Supplementary References

Supplementary Figure S1. ***B. subtilis*** **FtsZ N263K is PC190723-resistant.**

(**A**) Exponentially growing cells of strain DWA25 (*ftsZ cat*[FtsZ N263K]) were examined in the presence of either DMSO (1% v/v) or PC190723 (2 µg/mL), as indicated. Cell membranes were stained with FM5-95. Scale bar = 5 µm. (**B**) Strain DWA25 was streaked on NA + Cm in the absence and presence of PC190723 (2 µg/mL), as indicated. Plates were photographed after incubation at 37˚C for 18 h.

Supplementary Figure S1.

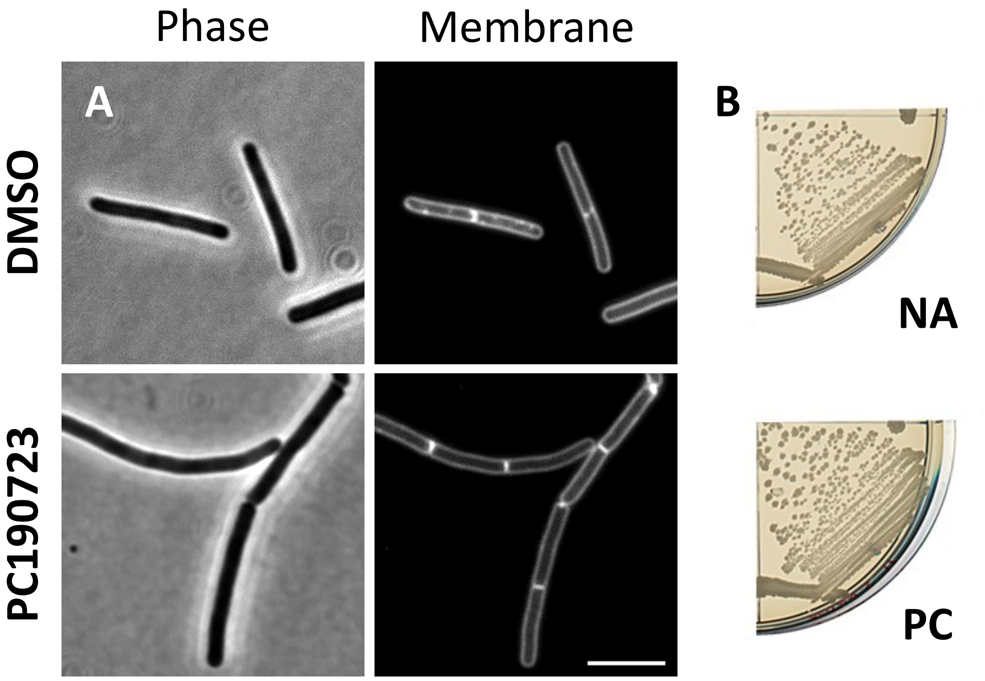

Supplementary Figure S2. ***S. aureus*** **strains R191P and G196A are 3-MBA-sensitive.**

S. aureus strains R191P, G193D, G196A, V214F, N263K and G266S were streaked on TSA in the absence and presence of either 3-MBA (10 mM) or PC190723 (8 µg/mL), as indicated. Plates were photographed after incubation at 37˚C for 18 h.

Supplementary Figure S2.

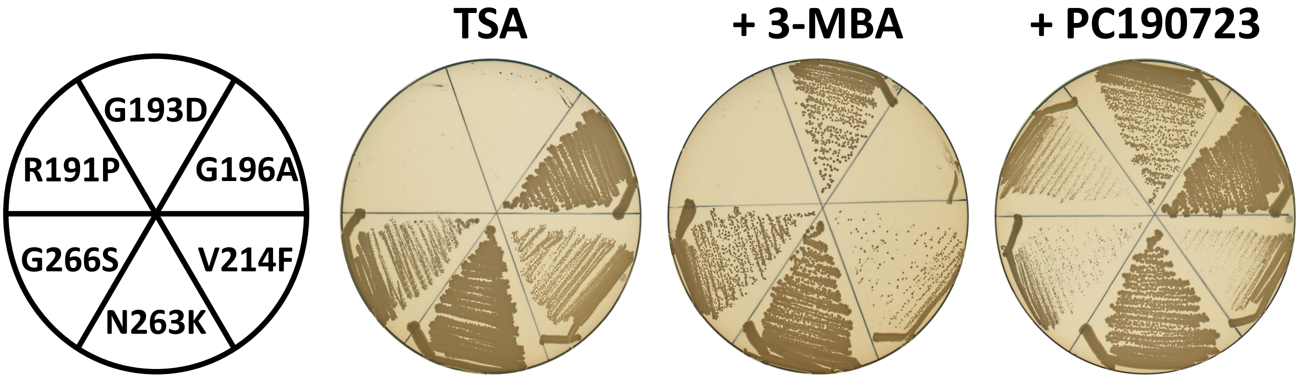

Supplementary Figure S3. Growth of benzamide-dependent and benzamide-independent strains of *B. subtilis*.

Strains (**A**) 168 (*ftsZ*[FtsZ WT]), (**B**) DWA454 (*ftsZ* sup9[FtsZ E251K]) and (**C**) DWA456 (*ftsZ* $sup9^{sup}$[FtsZ I228T, E251K]) were grown in LB at 37˚C in the presence of either DMSO (1% v/v) or 8J (2 µg/mL), as indicated. The optical density at 600 nm was measured at 60 min intervals and plotted against time.

Supplementary Figure S3.

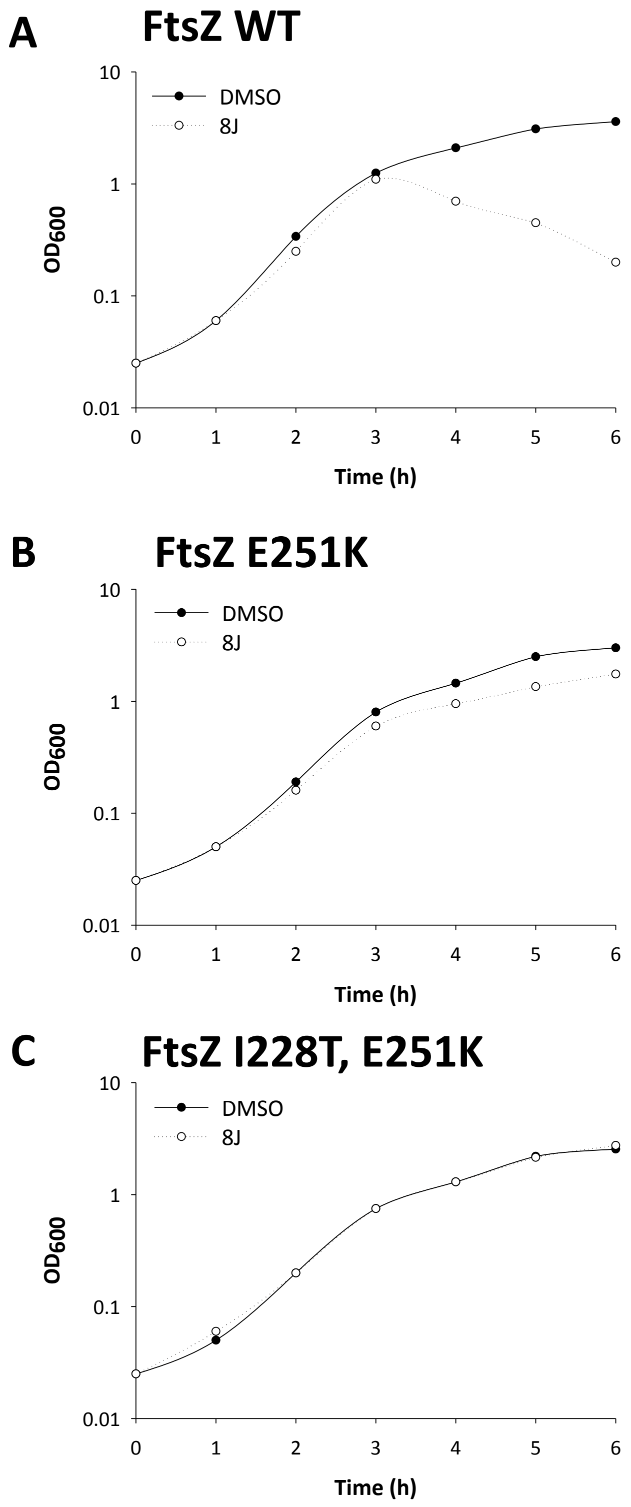

Supplementary Figure S4. Expanded field of view of benzamide-dependent cell division phenotype.

Exponentially growing cells of strain DWA454 (*ftsZ* sup9[FtsZ E251K]) were examined following growth in the presence of either DMSO (1% v/v) or 8J (8 µg/mL), as indicated. Cell membranes were stained with FM5-95. Scale bar = 5 µm.

Supplementary Figure S4.

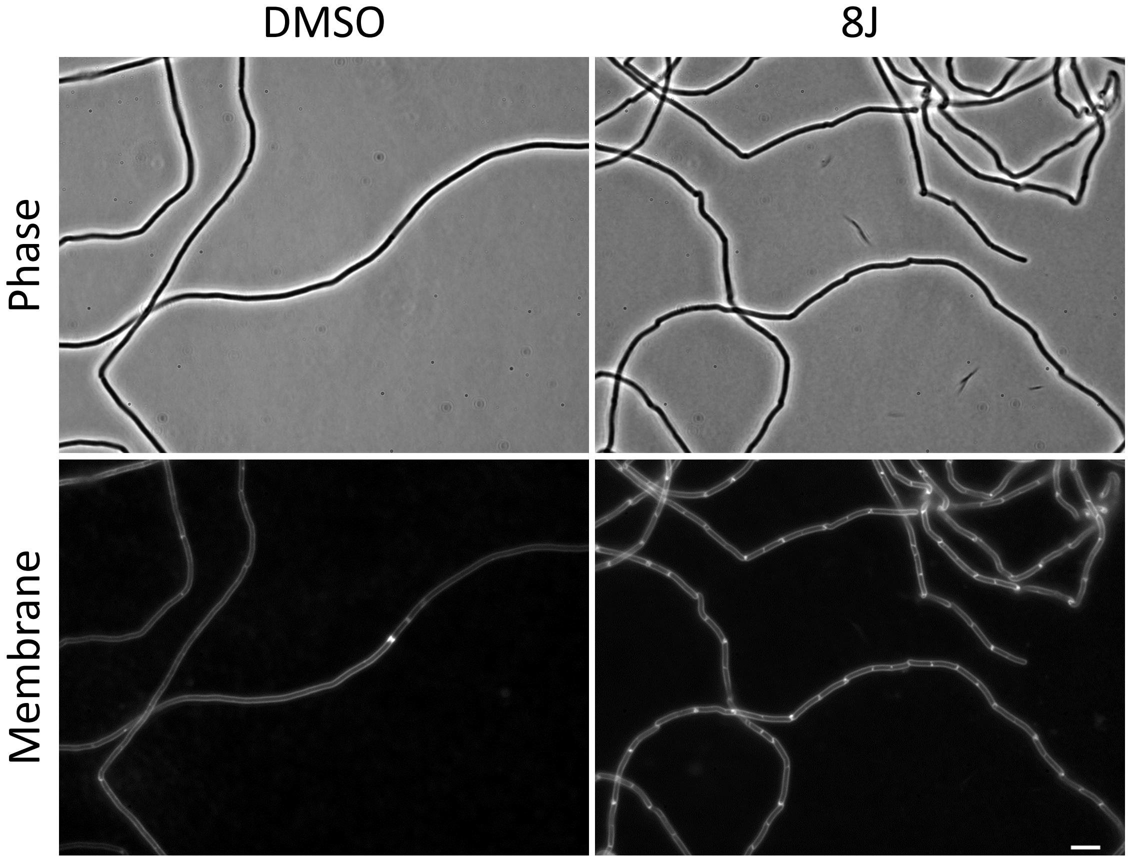

Supplementary Figure S5. 3-MBA-dependent division phenotype of FtsZ E251Q.

(**A**) Strain DWA30 (*ftsZ cat*[FtsZ E251Q]) was streaked on NA + Cm in the absence and presence 3-MBA (10 mM), as indicated. Plates were photographed after incubation at 37˚C for 18 h. (**B**) Exponentially growing cells of strain DWA30 were examined following growth in the presence of either DMSO (1% v/v) or 3-MBA (10 mM), as indicated. Arrowheads highlight unusual cell division events. Cell membranes were stained with FM5-95. Scale bar = 5 µm.

Supplementary Figure S5.

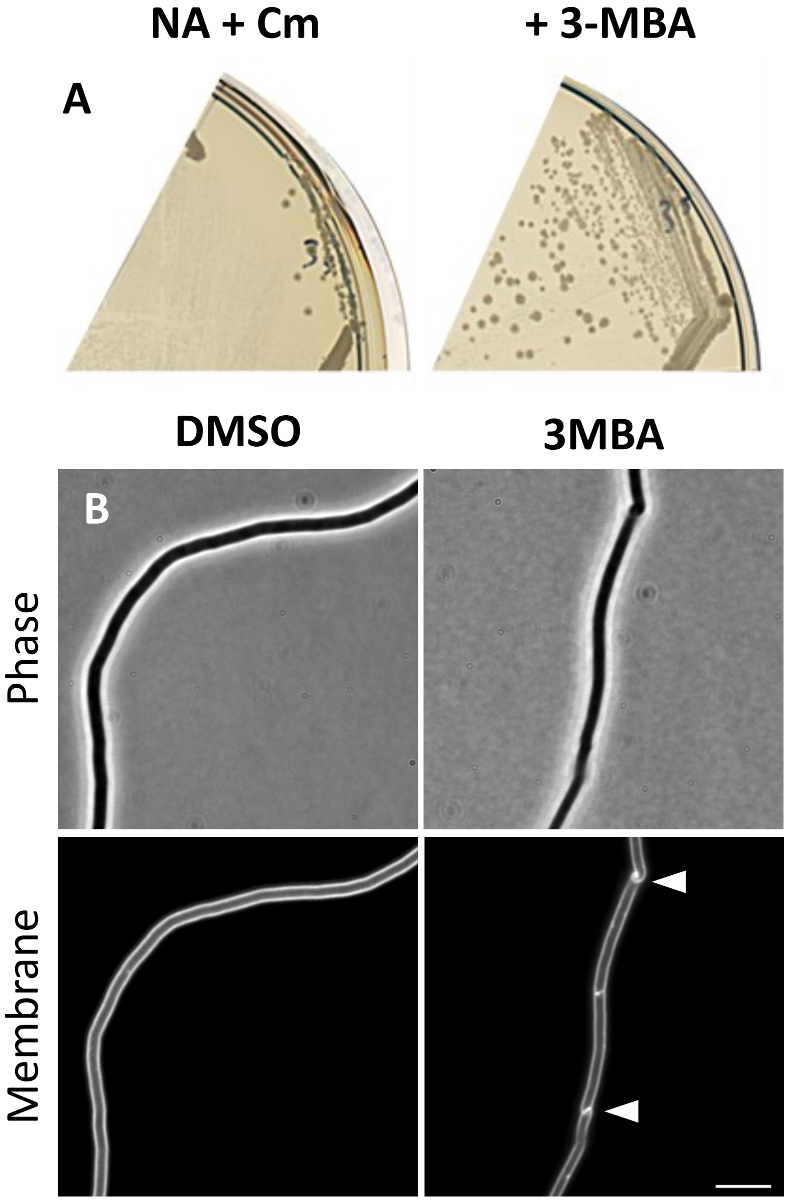

Supplementary Figure S6. Absence of helical cell division events in a Δ*ezrA* strain.

Exponentially growing cells of strains 168 and DWA14 (Δ*ezrA*::*spc*) were examined following growth for 1 h in the presence of either DMSO (1% v/v) or 8J (0.02 µg/mL), as indicated. Cell membranes were stained with Nile red. Scale bar = 5 µm.

Supplementary Figure S6.

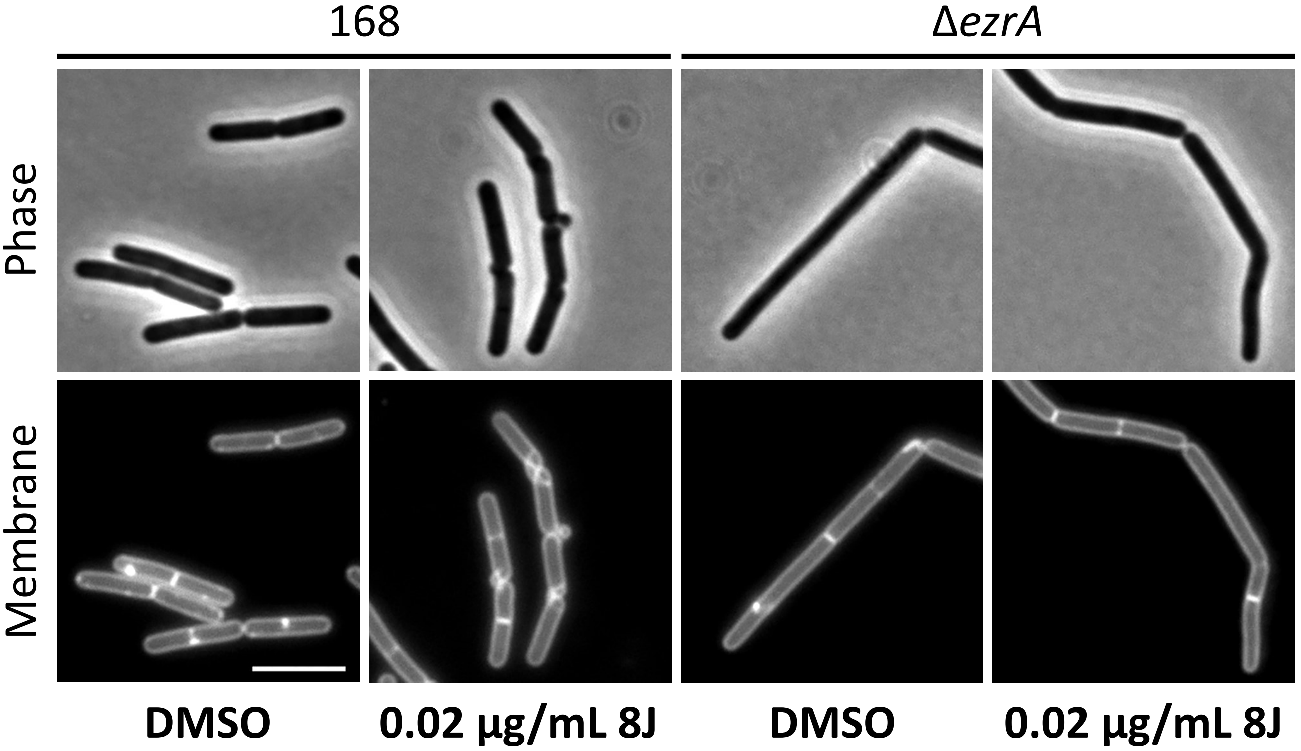

Supplementary Figure S7. FtsZ levels are comparable between *ftsZ* mutant strains.

Western blots showing the relative levels of FtsZ proteins (αFtsZ) produced under the control of the native promoter. All proteins are intact and are expressed at comparable levels. PBP2B (αPBP2B) levels are shown to control for sample loading. Cells were grown to exponential phase in LB at 37°C in the presence of either no additions, 1 % v/v DMSO (-), 8 µg/mL 8J (+) or chloramphenicol (+ Cm), as indicated, before being recovered and processed. Primary antibodies against FtsZ (Lucet *et al.*, 2000) and PBP2B (Daniel *et al.*, 2000) were used at a dilution of 1:10,000. Strains used: 168CA (WT parent), DWA454 (*ftsZ* sup9[FtsZ E251K]), DWA456 (*ftsZ* sup9^{sup}[FtsZ I228T, E251K]), DWA458 (*ftsZ cat*[FtsZ WT]), DWA402 (*ftsZ cat*[FtsZ I228V]), DWA414 (*ftsZ cat*[FtsZ I228V, E251K]), DWA401 (*ftsZ cat*[FtsZ I228L]) and DWA419 (*ftsZ cat*[FtsZ I228L, E251K]).

Supplementary Figure S7.

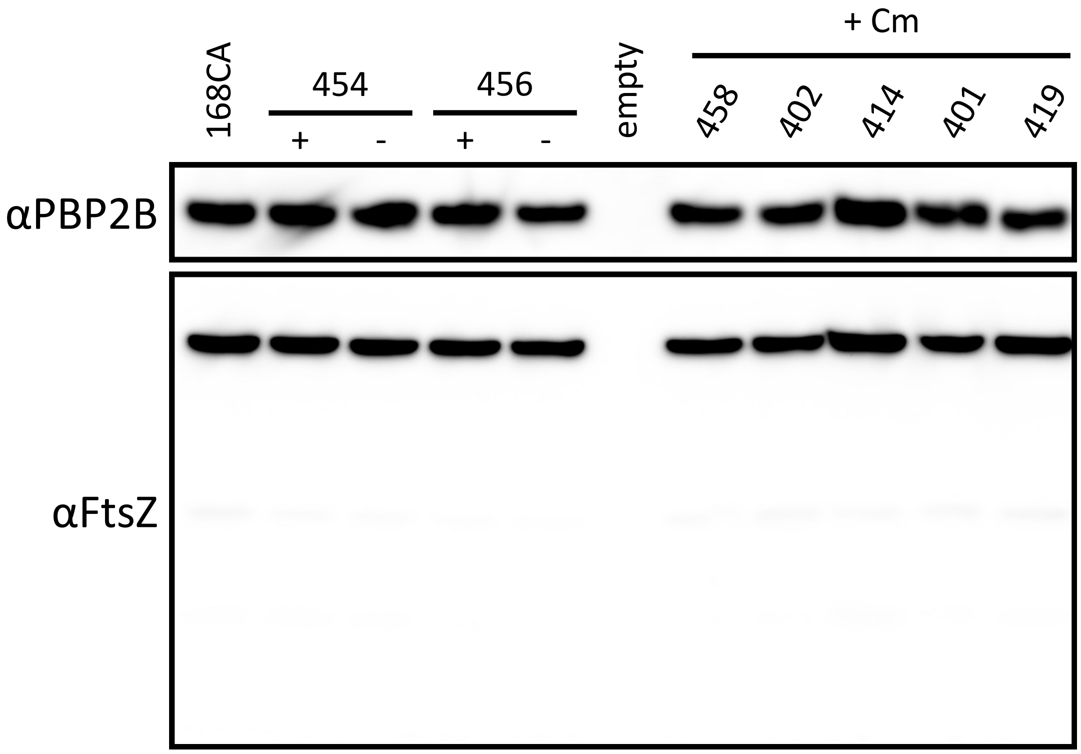

Supplementary Table S1. Bacterial strains

Strain	Relevant genotype[a]	Source/Construction[b]
B. subtilis		
168CA	*trpC2*	Laboratory stock
DWA14	*trpC2* Δ*ezrA*::*spc*	YK012>168CA (Sp)
DWA15	*trpC2* Δ*sepF*::*spc*	YK204>168CA (Sp)
DWA23	*trpC2 ftsZ sup1* (FtsZ G196S)	This work
DWA25	*trpC2* Ω*ftsZ*::[*ftsZ* (FtsZ N263K) *cat*]	pDWA8>168 (Cm, 8J)
DWA26	*trpC2* Ω*ftsZ*::[*ftsZ* (FtsZ G266S) *cat*]	pDWA9>168 (Cm, 3-MBA)
DWA27	*trpC2* Ω*ftsZ sup1*::[*ftsZ* (FtsZ G196S, N263K) *cat*]	pDWA8>DWA23 (Cm, 8J)
DWA28	*trpC2* Ω*ftsZ sup1*::[*ftsZ* (FtsZ G196S) *cat*]	pDWA8>DWA23 (Cm, 8J)
DWA29	*trpC2* Ω*ftsZ*::[*ftsZ* (FtsZ E251K) *cat*]	pDWA11>168 (Cm, 8J)
DWA30	*trpC2* Ω*ftsZ*::[*ftsZ* (FtsZ E251Q) *cat*]	pDWA12>168 (Cm, 3-MBA)
DWA33	*trpC2* Ω*ftsZ*::[*ftsZ* (FtsZ I228T, E251K) *cat*]	pDWA15>168 (Cm)
DWA35	*trpC2 ftsZ sup1* (FtsZ G196S), Δ*yshA-B*::*tet*	Sup1>1356 (8J)
DWA36	*trpC2 ftsZ sup1* (FtsZ G196S), Δ*noc*::*spc*	Sup1>1283 (8J)
DWA37	*trpC2 ftsZ sup1* (FtsZ G196S), Δ*sepF*::*spc*	Sup1>DWA15 (8J)
DWA38	*trpC2 ftsZ sup1* (FtsZ G196S), Δ*ezrA*::*spc*	Sup1>DWA14 (8J)
DWA39	*trpC2 ftsZ sup1* (FtsZ G196S), Δ*ugtP*::*spc*	Sup1>PG253 (8J)
DWA40	*trpC2 ftsZ sup1* (FtsZ G196S), Δ*minD*::*erm*	Sup1>1901 (8J)
DWA41	*trpC2 ftsZ sup9* (FtsZ E251K), Δ*yshA-B*::*tet*	Sup9>1356 (8J)
DWA42	*trpC2 ftsZ sup9* (FtsZ E251K), Δ*noc*::*spc*	Sup9>1283 (8J)
DWA43	*trpC2 ftsZ sup9* (FtsZ E251K), Δ*ezrA*::*spc*	Sup9>DWA14 (8J)
DWA44	*trpC2 ftsZ sup9* (FtsZ E251K), Δ*ugtP*::*spc*	Sup9>PG253 (8J)
DWA45	*trpC2 ftsZ sup9* (FtsZ E251K), Δ*minD*::*erm*	Sup9>1901 (8J)
DWA94	*trpC2* Δ*sepF*::*spc,* Ω*amyE*::(P_{xyl}-*sepF cat*)	YK204>168CA (Sp), >YK093 (Cm)
DWA401	*trpC2* Ω*ftsZ*::[*ftsZ* (FtsZ I228L) *cat*]	pDWA55>168CA (Cm)
DWA402	*trpC2* Ω*ftsZ*::[*ftsZ* (FtsZ I228V) *cat*]	pDWA52>168CA (Cm)
DWA410	*trpC2* Ω*ftsZ*::[*ftsZ* (FtsZ R168A) *cat*]	pDWA18>168CA (Cm)
DWA412	*trpC2* Ω*ftsZ*::[*ftsZ* (FtsZ E251A) *cat*]	pDWA13>168CA (Cm)
DWA414	*trpC2* Ω*ftsZ*::[*ftsZ* (FtsZ I228V, E251K) *cat*]	pDWA54>168CA (Cm/8J)
DWA419	*trpC2* Ω*ftsZ*::[*ftsZ* (FtsZ I228L, E251K) *cat*]	pDWA56>168CA (Cm/8J)
DWA454	*trpC2 ftsZ* sup9 (FtsZ E251K)	Sup9>168CA (8J)
DWA455	*trpC2* Ω*ftsZ*::[*ftsZ* (FtsZ E251K) *cat*]	DWA29>168CA (8J/Cm)
DWA456	*trpC2 ftsZ* sup9^{sup} (FtsZ I228T, E251K)	Sup9^{sup}>168CA (8J)
DWA458	*trpC2* Ω*ftsZ*::[*ftsZ* (FtsZ WT) *cat*]	pSG1928>168CA (Cm)
DWA459	*trpC2* Δ*ftsA*::*erm*	YK206>168CA (Em)
DWA460	*trpC2* Δ*sepF*::*spc*, Ω*amyE*::(P_{xyl}-*sepF cat*), *ftsZ* sup1 (FtsZ G196S)	Sup1>DWA94 (8J)
DWA461	*trpC2* Δ*sepF*::*spc*, Ω*amyE*::(P_{xyl}-*sepF cat*), *ftsZ* sup9 (FtsZ E251K)	Sup9>DWA94 (8J/0.5 % Xylose)
DWA637	*trpC2 ftsZ* sup9 (FtsZ E251K), Ω*aprE*::(*spc* P_{spac}-*yfp-zapA*)	PG67>168CA (Sp),>Sup9 (8J)
DWA638	*trpC2 ftsZ* sup9 (FtsZ E251K), Ω*aprE*::(*spc* P_{spac}-*yfp-ftsA*)	PG62>168CA (Sp),>Sup9 (8J)
DWA639	*trpC2 ftsZ* sup9 (FtsZ E251K), Δ*ftsA*::*erm*	YK206>DWA454 (Em, 8J)
DWA640	*trpC2 ftsZ* sup1 (FtsZ G196S), Δ*ftsA*::*erm*	YK206>DWA23 (Em, 8J)
YK012	CRK6000 Δ*ezrA*::*spc*	(Kawai & Ogasawara, 2006)
YK093	CRK6000 Ω*amyE*::(P_{xyl}-*sepF cat*)	(Ishikawa *et al.*, 2006)

YK204	CRK6000 Δ*sepF*::*spc*	(Ishikawa *et al.*, 2006)
YK206	CRK6000 Δ*ftsA*::*erm*	(Ishikawa *et al.*, 2006)
PG62	*trpC2* Ω*aprE*::(*spc* P_{spac}-*yfp-ftsA*)	(Gamba *et al.*, 2009)
PG67	*trpC2* Ω*aprE*::(*spc* P_{spac}-*yfp-zapA*)	(Gamba *et al.*, 2009)
PG253	*trpC2* Δ*ugtP*::*spc*	P Gamba, unpublished
1283	*trpC2* Δ*noc*::*spc*	(Wu & Errington, 2004)
1356	*trpC2* Δ*yshAB*::*tet*	(Feucht & Errington, 2005)
1901	*trpC2* Δ*minD*::*erm*	(Marston *et al.*, 1998)
S. aureus		
ATCC 29213	CLSI QC reference strain; MSSA	(Haydon *et al.*, 2008)
R191P	ATCC 29213, FtsZ R191P	(Haydon *et al.*, 2008)
G193D	ATCC 29213, FtsZ G193D	(Haydon *et al.*, 2008)
G196A	ATCC 29213, FtsZ G196A	(Haydon *et al.*, 2008)
V214F	ATCC 29213, FtsZ V214F	(Haydon *et al.*, 2008)
N263K	ATCC 29213, FtsZ N263K	(Haydon *et al.*, 2008)
RNpEzrA-CFP	RN4220 Ω*ezrA*::(*ezrA-cfp erm*)	(Pereira *et al.*, 2010)
DWA56	RN4220 Ω*ezrA*::(*ezrA-cfp erm*), FtsZ G196A	This Work

[a]Resistance gene abbreviations: *bla*, ampicillin; *cat*, chloramphenicol; *erm*, erythromycin; *spc*, spectinomycin; *tet*, tetracycline.

[b]For strains constructed by transformation, the source of the DNA used in the transformation is given first. The recipient strain is indicated after the arrow, with the selected marker in parentheses: Cm, chloramphenicol; Em, erythromycin; Sp, spectinomycin; Te, tetracycline.

Supplementary Table S2. Plasmids

Plasmid	Relevant genotype	Source/Construction
pSG1928	*bla cat ftsZ* (bp *4-1146*)	(Feucht & Errington, 2005)
pDWA6	*bla cat ftsZ* (FtsZ R191P)	This work
pDWA7	*bla cat ftsZ* (FtsZ G193D)	This work
pDWA8	*bla cat ftsZ* (FtsZ N263K)	This work
pDWA9	*bla cat ftsZ* (FtsZ G266S)	This work
pDWA11	*bla cat ftsZ* (FtsZ E251K)	This work
pDWA12	*bla cat ftsZ* (FtsZ E251Q)	This work
pDWA13	*bla cat ftsZ* (FtsZ E251A)	This work
pDWA14	*bla cat ftsZ* (FtsZ I228T)	This work
pDWA15	*bla cat ftsZ* (FtsZ I228T, E251K)	This work
pDWA18	*bla cat ftsZ* (FtsZ R168A)	This work
pDWA52	*bla cat ftsZ* (FtsZ I228V)	This work
pDWA54	*bla cat ftsZ* (FtsZ I228V, E251K)	This work
pDWA55	*bla cat ftsZ* (FtsZ I228L)	This work
pDWA56	*bla cat ftsZ* (FtsZ I228L, E251K)	This work

pDWA57	*bla cat ftsZ* (FtsZ I228A)	This work
pDWA58	*bla cat ftsZ* (FtsZ I228A, E251K)	This work
pDWA59	*bla cat ftsZ* (FtsZ I228G)	This work
pDWA60	*bla cat ftsZ* (FtsZ I228G, E251K)	This work

Supplementary Table S3. DNA oligonucleotides

Primer	Sequence 5'-3' (**mutation**)
R168A_F	GATCGTGATCCCGAACGAC**GC**TATCCTTGAAATTGTTGAT
R168A_R	ATCAACAATTTCAAGGATA**GC**GTCGTTCGGGATCACGATC
R191P_F	CGGATAACGTACTTC**C**CCAAGGGGTTCAAGG
R191P_R	CCTTGAACCCCTTGG**G**GAAGTACGTTATCCG
G193D_F	CGGATAACGTACTTCGCCAAG**AT**GTTCAAGGTATTTCTGACTTG
G193D_R	CAAGTCAGAAATACCTTGAAC**AT**CTTGGCGAAGTACGTTATCCG
I228A_F	CAAAGGATCTGCTTTGATGGGT**GC**CGGTATTGCTACTGGG
I228A_R	CCCAGTAGCAATACCG**GC**ACCCATCAAAGCAGATCCTTTG
I228G_F	CAAAGGATCTGCTTTGATGGGT**GG**CGGTATTGCTACTGGG
I228G_R	CCCAGTAGCAATACCG**CC**ACCCATCAAAGCAGATCCTTTG
I228L_F	AACAAAGGATCTGCTTTGATGGGT**C**TCGGTATTGCTAC
I228L_R	GTAGCAATACCGA**G**ACCCATCAAAGCAGATCCTTTGTT
I228T_F	ACAAAGGATCTGCTTTGATGGGTA**C**CGGTATTGCTACT
I228T_R	AGTAGCAATACCG**G**TACCCATCAAAGCAGATCCTTTGT
I228V_F	AACAAAGGATCTGCTTTGATGGGT**G**TCGGTATTGCTAC
I228V_R	GTAGCAATACCGA**C**ACCCATCAAAGCAGATCCTTTGTT
E251A_F	CCAGCCCGCTTCTTG**C**AGCGGCCATTGA
E251A_R	TCAATGGCCGCT**G**CAAGAAGCGGGCTGG
E251K_F	AGCAATTTCCAGCCCGCTTCTT**A**AAGCGGCCATTG
E251K_R	CAATGGCCGCTT**T**AAGAAGCGGGCTGGAAATTGCT
E251Q_F	CAATTTCCAGCCCGCTTCTT**CAG**GCGGCCATTGAC
E251Q_R	GTCAATGGCCGC**CTG**AAGAAGCGGGCTGGAAATTG
N263K_F	CAAGGCGTCCTCATGAA**G**ATCACTGGAGGAAC
N263K_R	GTTCCTCCAGTGAT**C**TTCATGAGGACGCCTTG
G266S_F	GGCGTCCTCATGAACATCACT**AGC**GGAACAAACCTCAG
G266S_R	CTGAGGTTTGTTCC**GCT**AGTGATGTTCATGAGGACGCC

Supplementary References

Daniel, R.A., E.J. Harry & J. Errington, (2000) Role of penicillin-binding protein PBP 2B in assembly and functioning of the division machinery of *Bacillus subtilis*. *Mol Microbiol* **35**: 299-311.

Feucht, A. & J. Errington, (2005) *ftsZ* mutations affecting cell division frequency, placement and morphology in *Bacillus subtilis*. *Microbiology* **151**: 2053-2064.

Gamba, P., J.W. Veening, N.J. Saunders, L.W. Hamoen & R.A. Daniel, (2009) Two-step assembly dynamics of the *Bacillus subtilis* divisome. *Journal of bacteriology* **191**: 4186-4194.

Haydon, D.J., N.R. Stokes, R. Ure, G. Galbraith, J.M. Bennett, D.R. Brown, P.J. Baker, V.V. Barynin, D.W. Rice, S.E. Sedelnikova, J.R. Heal, J.M. Sheridan, S.T. Aiwale, P.K. Chauhan, A. Srivastava, A. Taneja, I. Collins, J. Errington & L.G. Czaplewski, (2008) An inhibitor of FtsZ with potent and selective anti-staphylococcal activity. *Science* **321**: 1673-1675.

Ishikawa, S., Y. Kawai, K. Hiramatsu, M. Kuwano & N. Ogasawara, (2006) A new FtsZ-interacting protein, YlmF, complements the activity of FtsA during progression of cell division in *Bacillus subtilis*. *Mol. Microbiol.* **60**: 1364-1380.

Kawai, Y. & N. Ogasawara, (2006) *Bacillus subtilis* EzrA and FtsL synergistically regulate FtsZ ring dynamics during cell division. *Microbiology* **152**: 1129-1141.

Lucet, I., A. Feucht, M.D. Yudkin & J. Errington, (2000) Direct interaction between the cell division protein FtsZ and the cell differentiation protein SpoIIE. *EMBO J* **19**: 1467-1475.

Marston, A.L., H.B. Thomaides, D.H. Edwards, M.E. Sharpe & J. Errington, (1998) Polar localization of the MinD protein of *Bacillus subtilis* and its role in selection of the mid-cell division site. *Genes Dev.* **12**: 3419-3430.

Pereira, P.M., H. Veiga, A.M. Jorge & M.G. Pinho, (2010) Fluorescent reporters for studies of cellular localization of proteins in *Staphylococcus aureus*. *Appl Environ Microbiol* **76**: 4346-4353.

Wu, L.J. & J. Errington, (2004) Coordination of cell division and chromosome segregation by a nucleoid occlusion protein in *Bacillus subtilis*. *Cell* **117**: 915-925.
